# Is the reconstruction of medial support important for revision following failed treatment of femoral trochanteric fractures? a retrospective comparative study

**DOI:** 10.1186/s12891-022-06004-2

**Published:** 2022-11-29

**Authors:** Lin Qi, Wei Zhang, Zuhao Chang, Zhaoxia Zuo, Jiaqi Li, Jiantao Li, Hua Chen, Peifu Tang

**Affiliations:** 1 Chinese PLA Medical School, No.28 Fuxing Road, Haidian, Beijing, 100853 China; 2 Department of Orthopaedics, the First Medical Center, Chinese PLA General Hospital, No.28 Fuxing Road, Haidian, Beijing, 100853 China; 3 School of Sports Engineering, Beijing Sport University, No. 48 Information Road, Haidian District, Beijing, 100084 China; 4 National Clinical Center for Orthopedics, Sports Medicine & Rehabilitation, No.28 Fuxing Road, Haidian, Beijing, 100853 China

**Keywords:** Trochanteric fracture, Nonunion, Implant failure, Medial support, Revision

## Abstract

**Background:**

Hip-preserving revision in patients with failed treatment of femoral trochanteric fracture is still a major challenge. Whether the medial support reconstruction could benefit the patients and improve the success rate of hip-preserving revision is still controversial. Hence, the purpose of this study was to evaluate the clinical significance and prognosis of medial support reconstruction during the hip-preserving revision of failed femoral trochanteric fracture treatment.

**Methods:**

Patients with failed femoral trochanteric fractures treatments addressed by hip-preserving revision at our hospital from January 2014 to December 2020 were analyzed retrospectively. 31 patients were included and divided into a medial support group (*n* = 16) and a non-medial support group (*n* = 15). The fracture healing rate was the primary measurement. In addition, the differences in Oxford Hip Score (OHS), quality of life, surgical trauma, and complications were also evaluated.

**Results:**

The fracture healing rate (100%, 16/16 vs. 66.67%, 10/15), the OHS (42.06 ± 4.12 vs. 30.93 ± 11.56, M ± SD), and the mental component score of the 12-item Short-Form Survey (SF-12) (54.48 ± 5.38 vs. 47.90 ± 3.47, M ± SD), were significantly better and the incidence of complications [0(0/16) vs. 40%(6/15)] was significantly lower in the medial support group than the non-medial support group (*p* < 0.05). No significant differences in the physical component score of the SF-12, surgical trauma and reduction in collodiaphyseal angle of affected femur were observed between groups.

**Conclusions:**

The reconstruction of medial support seems important for revision following failed treatment of femoral trochanteric fractures. Due to the medial augmentation and improvement of the mechanical stability for proximal femur, the patients might benefit from fracture healing prognosis and functional.

## Background

Femoral trochanteric fracture is a severe public health problem, usually treated surgically [1–4]. However, due to the complexity of fracture patterns, failure after operation could not be avoided [1, 5], leading to enhanced dysfunction and increased mortality. Therefore, a revision operation represents an essential and necessary option for patients, who experience surgical failure of trochanteric fracture, to improve capacity to live independently [6]. For physiologically young patients with long life expectancies and adequate bone quality for fixation, hip-preserving revision is the more recommended treatment [1, 7, 8]. However, which hip-preserving fixation is more effective and stable remains controversial [1, 5, 9].

The single intramedullary or extramedullary fixation system remains the most common hip-preserving surgical approach [1, 2, 6]. However, inherent design flaws associated with the implants and the complex biomechanical environment of the proximal femur still be remained, and this revision strategy has the similar potential risk of the surgical failure. [10, 11]. Even when combined with a valgus osteotomy, single internal fixation approaches have obvious disadvantages, including abnormal alignment and degeneration of the affected limb joints [12, 13].

Hence, the reconstruction of the medial support was introduced to hip-preserving treatment, combining an anterior medial plate with the single intramedullary or extramedullary implant, which provides medial support for the proximal femur, to increases the mechanical stability of the nonunion site, and improve the success rate of revision operation [1, 9, 10]. However, due to additional procedure of the medial support implant, the augmentation strategy might increase surgical trauma, longer operation times, result in more complications, and negatively effect on the limb function [14]. To further evaluate the clinical significance and prognosis associated with medial support reconstruction in hip-preserving revisions for failed femoral trochanteric fracture treatment, we conducted a retrospective comparative study to examine fracture healing rate, functional outcome, quality of life, surgical trauma, and complications between groups with and without medial support reconstruction in revision treatment.

## Methods

### Data source

This study was a single-center retrospective case–control study. We reviewed the electronic medical records and radiologic data of patients diagnosed with nonunion of femoral trochanteric zone and were treated with hip-preserving revision surgery at our hospital from January 2014 to December 2020. Patients were followed up to assess functional and quality of life outcomes during outpatient clinic visits or telephone. The study was approved by the medical ethics committee and we have obtained a comprehensive agreement for academic use of information during their treatments from the patients at the time of their hospitalization.

### Inclusion and exclusion criteria

Inclusion criteria: (1) Initial operation failure: ①Implant failure, including cutout or backing out of the cephaloscrew, fracture of the nail or plate, or obvious loss of reduction (varus > 10°, rotation > 15°); ②Nonunion, in which the fracture failed to heal 6 months postoperatively with no signs of further healing on imaging after nearly 3 months; (2) hip-preserving revisions were performed; (3) follow-up for at least 12 months.

Exclusion criteria: (1) age < 18 years; (2) acute or chronic osteomyelitis; (3) soft tissue infection; (4) avascular necrosis of the femoral head; (5) severe hip osteoarthritis (Kellgren–Lawrence Grade 4); (6) pathological fracture; (7) segmental bone defect ≥ 6 cm; (8) death; and (9) poor compliance.

### Grouping

Following inclusion and exclusion criteria, 40 patients with failed trochanteric fracture treatment were identified. Among them, 6 patients were lost to follow-up, and 3 died. Finally, 31 patients, including 23 men and 8 women, were included in the study, with an average age of 57.48 ± 16.48 years (range: 23–86 years). These patients were divided into two groups: (1) the medial support group containing 16 cases and (2) the non-medial support group containing 15 cases. All patients received autogenous bone grafts (ABGs).

Among the 16 patients in the medial support group, 9 patients required the replacement of the original implant, 7 patients retained their original implant. All patients were treated with an open reduction and internal fixation. Based on the anatomical and mechanical characteristics of the proximal femur, a cephalomedullary nail, 5.0-mm locking plate, or 95° dynamic condylar screw (DCS) (ZhengTian Medical Instrument Co. Ltd, Tianjin, China) was used to reconstruct the lateral wall; and a medial anatomic buttress plate (MABP) [15] (ZhengTian Medical Instrument Co. Ltd, Tianjin, China) was placed on the anterior medial side of the proximal femur to reconstruct the medial support.

Among the 15 patients in the non-medial support group, 8 patients received a replacement implant using a single intramedullary or extramedullary fixation system to reconstruct the lateral wall, 7 patients retained their original implant.

### Operative technique

Under general anesthesia, the patient was placed in the supine position on a radiolucent operating table.

Different surgical approaches and methods were adopted according to whether the primary implants were replaced or not. (1) If the primary implant needs to be replaced, use the Watson-Jones approach. The primary internal fixation was removed, the fibroconnective tissues at the fracture were removed with a rongeur, and the sclerotic bone in the fracture ends was ground with a grinding drill. The Collodiaphyseal Angle(CDA) was restored with traction of the affected limb and fixed with a cephalomedullary nail, 5.0-mm locking plate, or DCS with the monitoring using fluoroscopy. The structural bone grafts and bone paste, taken from the anterior iliac crest, was placed into the nonunion site. Then, at the level of the lesser trochanter of the medial proximal femur, the MABP was used to reconstruct the medial support of the proximal femur. Finally, close the deep fascia, subcutaneous tissue and skin separately. (2) If the primary internal fixation is retained, the anterior approach of proximal femur (distal extension of the Smith-Petersen approach) [16] is adopted. After debridement of nonunion site and autologous bone grafting, the MABP was placed at the level of the lesser trochanter of the medial proximal femur to reconstruct the medial support. Finally, close the wound layer by layer.

The difference between the medial support group and the non-medial support group is whether to use MABP to reconstruct the medial support of proximal femur. All patients underwent debridement and autologous bone grafting of nonunion site.

### Postoperative rehabilitation and follow-up

On postoperative day one (PD1), all patients were allowed full-range functional exercises, without weight-bearing, to avoid joint stiffness, and isometric contractions were performed to prevent muscle atrophy. Radiographic examinations were used to evaluate the quality of fracture reduction and the position of internal fixation. Functional and radiological evaluations were performed postoperatively at outpatient clinics. Only after the fracture is healed should the patient perform complete weight-bearing activities.

### Evaluation criteria

The primary outcome measurement was fracture healing rate. The criteria for fracture healing were as follows: ①imaging criteria: X-ray shows a blurred fracture line, and at least three of four cortices have bridging callus passing through; or CT shows a continuous bridging callus passing through 25% of cortices on transverse section; ②clinical criteria: no tenderness during the local examination, and no pain when walking with full weight-bearing. These two criteria must be met simultaneously. Fracture healing can only be diagnosed by interobserver agreement. If fracture healing cannot be confirmed by X-ray, CT scan should be obtained. Radiological assessments were performed by two senior authors.

Secondary outcome measurements were as follows:

1. Oxford Hip Score (OHS): The scale was performed by telephone or outpatient follow-up. Patients are followed up by two authors at intervals of two weeks to avoid bias associated with both the patient and the author. The average of the results of these two follow-ups was taken as the results of the last follow-up. The scale evaluation criteria are as follows: Excellent: 40–48; Good: 30–39; General: 20–29; Poor: 0–19.
2. General Health Outcomes: Physical Component Score of the 12-item Short-Form Survey (PCS-12), and Mental Component Score of the 12-item Short-Form Survey (MCS-12), the follow-up and bias control method of the patient-rated outcome measure (PROM) are similar to OHS. And take 37.5 points as the tiering criterion (a score lower than 25% of the U.S. general population average score) [17];
3. Surgical Trauma: Incision length (in cm), red blood cell (RBC) transfusion (in U), operation time (in min);
4. Complications: reduction in the collodiaphyseal angle (in °), implant failure, poor wound healing, malunion, nonunion, soft tissue or bone infection, joint stiffness, neurovascular injury, periprosthetic fracture, avascular necrosis of the femoral head, deep venous thrombosis, pulmonary embolism, and reoperation rate (%).

All data were collected by three orthopedic residents not involved in the treatment of patients**.**

### Data analysis

All data were analyzed by SPSS v.25 (IBM Corp, Armonk, New York). Quantitative data with normal distribution and homogeneity of variance are expressed as the mean ± standard deviation (M ± SD). Other data are expressed as the median (quartile 1, quartile 3) [M (Q1, Q3)]. Comparisons of quantitative data between two groups were performed by independent-samples t-test for parametric variables or the Mann–Whitney U test for non-parametric variables. Qualitative data are expressed in numbers and compared between groups using the Chi-square test or Fisher’s Exact test. A *p*-value < 0.05 was considered significant.

## Results

**Fig. 1:**
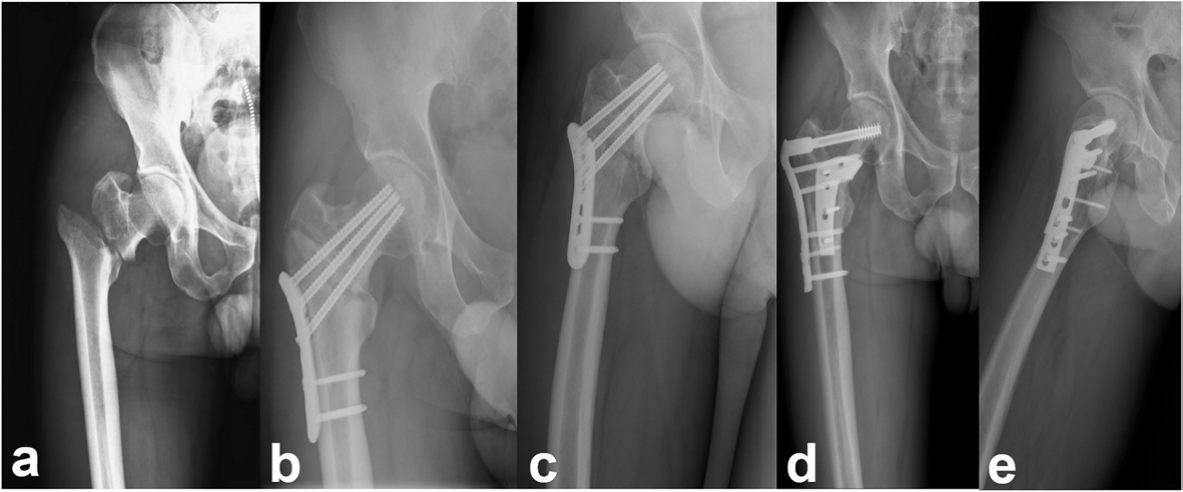
A 41-year-old man with right trochanteric fracture (AO/OTA A2) initially fixed by proximal femoral locking plate. **a** The X-ray of original fracture. **b**-**c** Nine months after surgery, X-rays showed fracture not yet healed. **d**-**e** The previous implant was replaced with a DCS and a MABP. **d** The X-ray showed the fracture healed three months after revision surgery

**Fig. 2:**
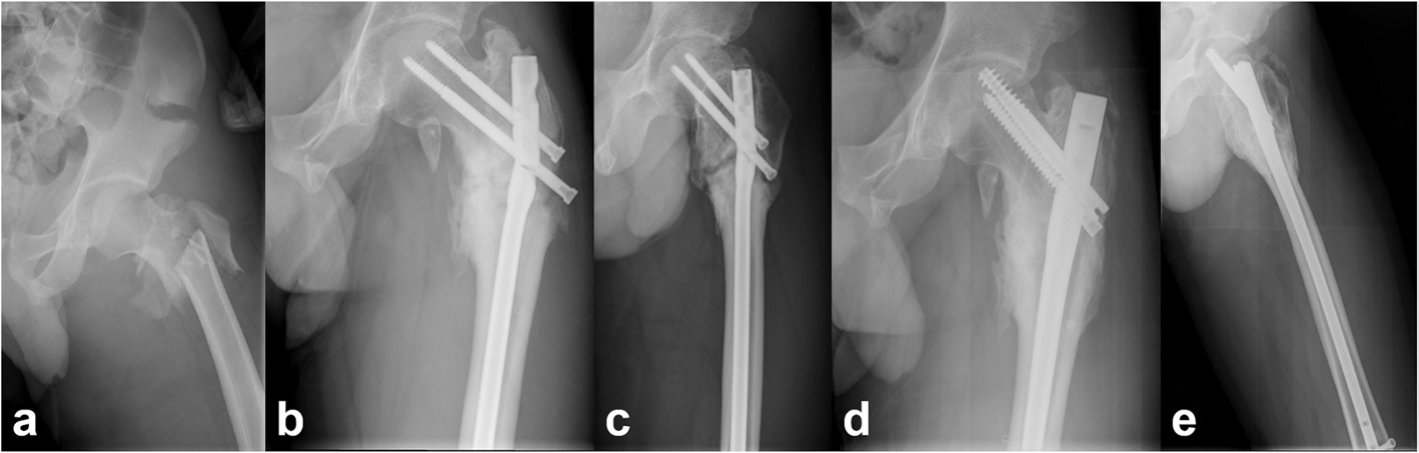
A 23-year-old man with left trochanteric fracture (AO/OTA A3) was initially fixed by a long femoral reconstruction intramedullary nail. **a** The X-ray of original fracture. **b**-**c** The initial implant broke ten months after the operation with the fractur nonunion. **d**-**e** The previous implant was replaced with an InterTAN without medial support reconstruction, and the fracture healed 6 months after revision surgery

Two senior doctors analyzed the patients' clinical and demographic characteristics, with no significant differences identified between the two groups (Table 1). The mean follow-up times were 29.63 ± 16.75 months in the medial support group and 42.87 ± 19.87 months in the non-medial support group, which was not significantly different. Typical cases are shown in Figs. 1 and 2.

**Table 1:** The demographic characteristics of the patients

	**Medial support group** (*n* = 16)	**Non-medial support group** (*n* = 15)	*P*-value*
Sex (n)			1
Male/female	11/5	11/4	
Age (y)	55.8 ± 11.9	60.5 ± 20.3	0.4362
BMI (kg/m^2^)	24.6 ± 3.8	23.8 ± 3.4	0.586
Cause of injury (n)			0.1556
Low energy/high energy	6/10	10/5	
Time since injury (months)	9.5 (7, 13.3)	8 (4.5, 13)	0.5005
Classification of original fracture			0.373
31A1	4	3	
31A2	2	5	
31A3	10	7	
ASA classification (n)	1.5 (1, 2)	2 (1, 2)	0.5539
1/2/3	8/6/2	5/9/1	
Singh Index Rating (n)	4.5 (3, 6)	4 (3, 5)	0.4058
1/2/3/4/5/6	0/0/5/3/3/5	0/3/3/2/4/3	
aCCI	2 (0, 4)	4 (1, 4.5)	0.2747
Loss of medial support (n)	10	12	0.4331
Varus deformities (n)	12	15	0.1012

^*^Independent-samples t-test or Mann–Whitney U test for quantitative data, Chi-square test or Fisher’s Exact test for qualitative data

*BMI *Body Mass Index, *ASA *American Association of Anesthesiologists, *aCCI* Age-Corrected Charlson Complications Index

### Primary outcome

The fracture healing rate were significantly better in the medial support group than the non-medial support group (Table 2).

**Table 2:** Clinical outcomes

	**Medial support group** **(*n* = 16)**	**Non-medial support group** **(*n* = 15)**	*P*-value*
FHR (%)	100(16/16)	66.67(10/15)	0.042
OHS	42.06 ± 4.12	30.93 ± 11.56	0.0040
PCS-12	45.45 ± 5.55	42.69 ± 7.00	0.1880
MCS-12	54.48 ± 5.38	47.90 ± 3.47	0.0001

*OHS* Oxford Hip Score, *PCS-12* Physical Component Score-12, *MCS-12* Mental Component Score-12, *FHR* Fracture healing rate

^*^Independent-samples t-test for parametric variables or Mann–Whitney U test for non-parametric variables

### Secondary outcome

#### Oxford hip score

Compared with the non-medial support group, the OHS was significantly better in the medial support group (Table 2). In the medial support group, 12 patients had excellent OHS, 4 were Good, compared with 4 excellent, 4 Good, 5 General, and 2 Poor values in the non-medial support group, and a significant difference was identified between the two groups (*p* = 0.012).

#### General health

The MCS-12 was significantly better in the medial support group (Table 2). For the PCS and MCS of SF-12, patients in the medial support group with a score < 37.5 accounted for 12.5% (2/16) and 0 (0/16), respectively. In the non-medial support group, 26.7% (4/15) and 6.7% (1/15).

#### Surgical trauma

The groups noted no significant differences in incision length, RBC transfusion and operation time (Table 3).

**Table 3:** Surgical trauma and the CDA of affected side

	**Medial support group** **(*n* = 16)**	**Non-medial support group** **(*n* = 15)**	*P*-value*
Incision length (cm)	15 (9.5, 21.3)	10 (4.5, 20)	0.3482
RBC transfusion (U)	5.72 ± 4.49	5.40 ± 3.09	0.8000
Operation time (min)	214.13 ± 65.67	206.67 ± 84.59	0.7110
Preoperative CDA (°)	113.68 ± 9.51	108.65 ± 15.45	0.3580
Postoperative CDA (°)	125.36 ± 7.55	129.29 ± 8.59	0.1190

*RBC* Red blood cell, *CDA* Collodiaphyseal Angle

^*^Independent-samples t-test for parametric variables or Mann–Whitney U test for non-parametric variables

#### Complications

**Fig. 3:**
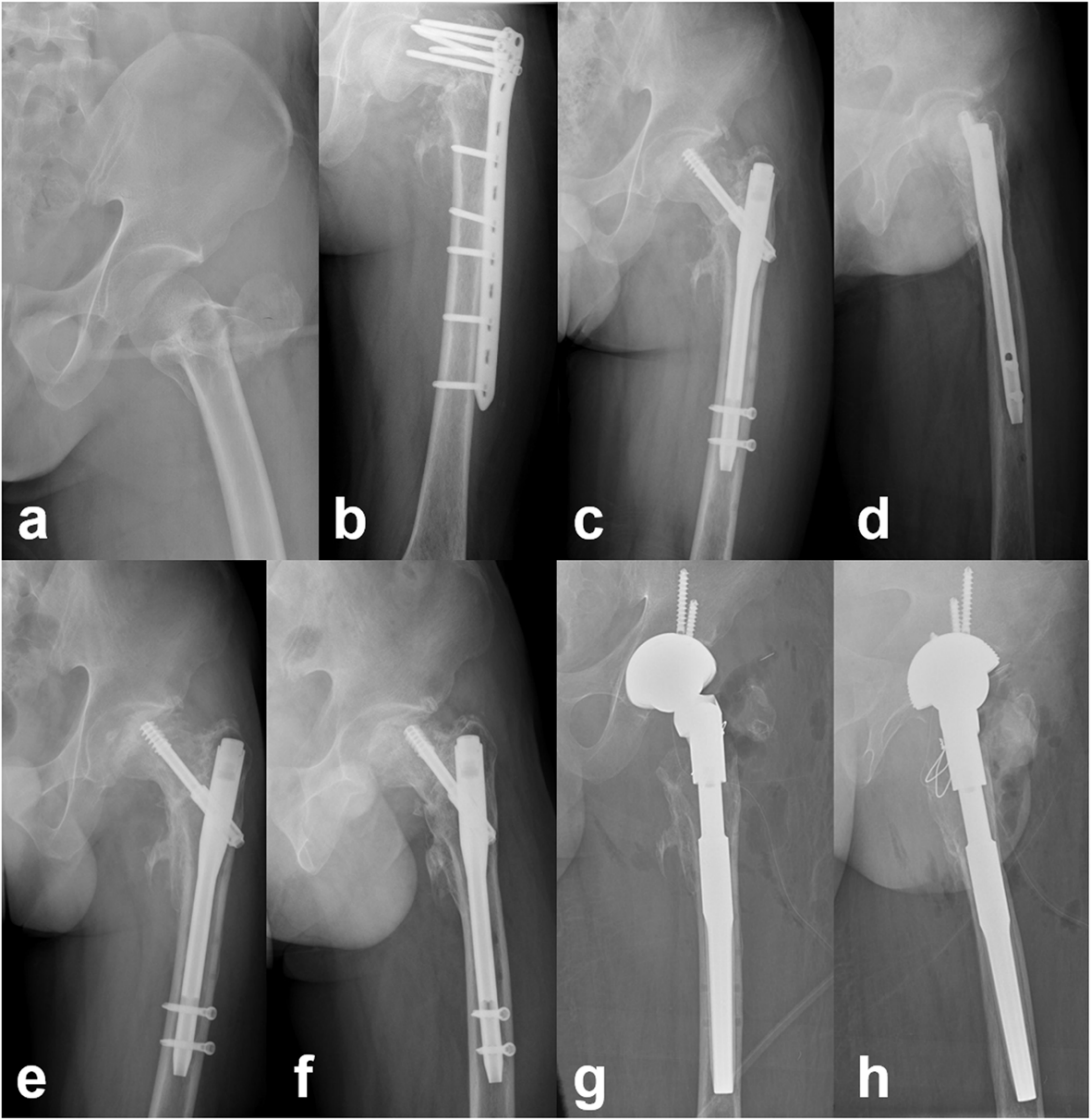
A 42-year-old man with left trochanteric fracture (AO/OTA A3) initially fixed by inverted less invasive stabilization system plate. **a** The X-ray of original fracture. **b** Ten months postoperatively, X-ray showed implant failure with varus displacement and nonunion. **c**-**d** The previous implant was replaced with a Gamma nail without medial support reconstruction, and the fracture healed nine months after the revision surgery. **e**–**f** Twelve months after the revision surgery, X-ray showed avascular necrosis of the femoral head. **g**-**h** Twenty-one months after the revision surgery, THA was used as salvage treatment

During follow-up, no complications were reported in the medial support group, whereas complications occurred in 6 cases in the non-medial support group [0(0/16) vs. 40% (6/15), *p* = 0.018]: (1) five cases underwent total hip arthroplasty (THA) due to implant failure with nonunion and obvious varus deformity; and (2) one case underwent total hip arthroplasty (THA) due to osteonecrosis of the femoral head, combined with cephaloscrew cutout. (Fig. 3).

## Discussion

The study showed that (1) the healing rate was relatively higher for the medial support group than for the non-medial support group; (2) the OHS, and MCS-12 of the medial support group were significantly better than those of the non-medial support group; (3) the incidence of complications was lower for the medial support group, but no significant differences were observed for surgical trauma. Therefore, the reconstruction of medial support may be important for revisions of failed femoral trochanteric fracture treatments and might improve clinical prognosis.

At present, single intramedullary or extramedullary fixation are still used in many hip-preserving operations most commonly angle-stable plate, cephalomedullary nail, or locking compression plate (LCP) [1, 2, 6, 18]. However, single implant for revision fixation is often unable to achieve a satisfactory clinical prognosis. Lambers et al. [19] revised 11 patients with failed trochanteric fracture treatment by replacing the cephalomedullary nail, resulting in a failure rate as high as 27.3%. Another commonly used fixator, dynamic hip screw (DHS), must be combined with a valgus osteotomy to achieve a relatively high healing rate. [12, 13] However, this procedure alters the normal alignment of the lower extremities, increasing the risks of joint pain and degeneration and significantly increasing the difficulty of further salvage revisions [12]. Lotzien et al. [10, 20] did not use valgus osteotomy but applied DCS as the primary fixation method for treating patients following surgical failure of intertrochanteric fractures. The failure rate of revision surgery among the DCS group was 44.4% (4/9) [12].

In response to the above problems, the value of the medial support reconstruction of the proximal femur has gradually attracted surgeons’ attention. Finite element analysis and biomechanical studies have shown that the integrity of the medial wall is important for increasing the destructive load. Furthermore, compared with the reconstruction of the integrity of lateral wall in proximal femur, the integrity of the medial wall may be taken priority during the fracture fixation to achieve better stability. [21, 22]. Relevant clinical studies have pointed out that poor restoration of the medial wall of the femur will make the intramedullary nail fixation tend to fail [23]. Xue et al. [9] also found the importance of restoring effective medial support at the intertrochanteric region in the treatment of intertrochanteric fracture nonunion.

Therefore, given the potential benefits associated with the reconstruction of medial support, MABP, as a specific implant that restores the medial support of the hip, has been designed and successfully applied to the treatment of femoral neck fracture and nonunion [24, 25]. Hence, to furtherly evaluate the importance of medial support reconstruction in hip-preserving revision surgery following failed treatment of inter/peri-trochanteric fractures, we conducted the present study. The clinical prognosis of patients in medial support group in our study showed better fracture healing outcome, higher functional scores, and less complications than ones in non-medial support group and the other previous related studies [9, 10, 20], which were contributed by better improvement of mechanical and biological environment on nonunion site simultaneously: (1) Direct medial support: Previous medial support plates are placed anterior to the proximal femur, only provide indirect support [9, 10]. While, MABP is placed just below the femoral neck and medial side of proximal femur to provide direct support. This placement position of MABP can better abolish the varus stress of the proximal femur, and provide more effective mechanical stability for nonunion site. This advantage was also preliminarily confirmed by biomechanical studies, which might benefit patients in terms of early functional exercises and fracture healing [26]; (2) Thorough debridement and autograft: The intertrochanteric region is rich in blood supply and cancellous bone. Hence, when revision is carried out, it is previously believed that thorough debridement and autograft of the nonunion site are not necessary [27]. However, through our study, it was found that the success rate of fracture healing and one-stage revision surgery in both groups were significantly higher than previous clinical reports. This is most likely related to thoroughly debriding and autografting in all patients. However, it is worth noting that the failure rate of revision surgery remained 66.7% higher in the non-medial support group, and the insufficient improvement of mechanical stability carried by the single implant may contribute to this outcome.

However, this study also has some limitations. First, as a retrospective comparative study, this study is vulnerable to the inherent inclusion and exclusion biases that cannot be adjusted. Second, the relatively small patient sample size and improper control of confounding factors may affect the corresponding conclusions. Finally, a lack of consistency exists between implant species and fixation constructions. Although these differences in implant selection followed basic principles and standard surgical procedures were applied to treat nonunion, they remain associated with stability differences in the final fixation.

## Conclusions

In summary, hip-preserving revision remains the first-line option for physiologically young patients with failed femoral trochanteric fracture treatment. Among patients who underwent the reconstruction of medial support, fracture healing occurred at a higher rate due to the enhanced stability of the nonunion site compared with those who did not receive medial support reconstruction. Moreover, compared with single intra- or extramedullary fixation, the reconstruction of medial support in the trochanteric area is likely beneficial for patient health outcomes. Finally, THA might represent a more reasonable choice for patients with a failed hip-preserving revision as a salvage option. Surgical failures among older patients with trochanteric fractures may become more common with the increasingly aging population. High-quality, prospective clinical studies remain necessary to confirm whether the reconstruction of medial support can optimize clinical prognosis.

## Data Availability

The datasets used and/or analyzed during the current study are available from the corresponding author on reasonable request.

## Abbreviations

OHS: Oxford Hip Score
SF-12: The 12-item Short-Form Survey
ABGs: Autogenous bone grafts
LCP: Locking compression plate
DCS: Dynamic condylar screw
MABP: Medial anatomic buttress plate
PD1: Postoperative day one
PCS-12: Physical Component Score of the 12-item Short-Form Survey
MCS-12: Mental Component Score of the 12-item Short-Form Surve
PROM: Patient-rated outcome measure
RBC: Red blood cell
M ± SD: Mean ± standard deviation
M (Q1, Q3): Median (quartile 1, quartile 3)
FHR: Fracture healing rate
MOS: Months
CDA: Collodiaphyseal Angle
THA: Total hip arthroplasty
DHS: Dynamic hip screw

## References

[1] Liu P, Jin D, Zhang C, Gao Y (2020). Revision surgery due to failed internal fixation of intertrochanteric femoral fracture: current state-of-the-art. BMC Musculoskelet Disord.

[2] Dziadosz D (2015). Considerations with failed intertrochanteric and subtrochanteric femur fractures: how to treat, revise, and replace. J Orthop Trauma.

[3] Johnell O, Kanis JA (2006). An estimate of the worldwide prevalence and disability associated with osteoporotic fractures. Osteoporos Int.

[4] Leer-Salvesen S, Engesæter LB, Dybvik E, Furnes O, Kristensen TB, Gjertsen JE (2019). Does time from fracture to surgery affect mortality and intraoperative medical complications for hip fracture patients? An observational study of 73 557 patients reported to the Norwegian Hip Fracture Register. Bone Joint J.

[5] Kaplan K, Miyamoto R, Levine BR, Egol KA, Zuckerman JD (2008). Surgical management of hip fractures: an evidence-based review of the literature. II: intertrochanteric fractures. J Am Acad Orthop Surg.

[6] Babcock S, Kellam JF (2018). Hip Fracture Nonunions: Diagnosis, Treatment, and Special Considerations in Elderly Patients. Adv Orthop.

[7] Haidukewych GJ, Berry DJ (2005). Salvage of failed treatment of hip fractures. J Am Acad Orthop Surg.

[8] Haidukewych GJ, Berry DJ (2003). Salvage of failed internal fixation of intertrochanteric hip fractures. Clin Orthop Relat Res.

[9] Xue D, Yu J, Zheng Q, Feng G, Li W, Pan Z (2017). The treatment strategies of intertrochanteric fractures nonunion: An experience of 23 nonunion patients. Injury.

[10] Lotzien S, Rosteius T, Rausch V, Schildhauer TA, Geßmann J (2020). Trochanteric femoral nonunion in patients aged over 60 years treated with dynamic condylar screw. Injury.

[11] Marecek G, Centomo H (2019). Augmented Fixation for Fractures of the Appendicular Skeleton. J Am Acad Orthop Surg.

[12] Said GZ, Farouk O, El-Sayed A, Said HG (2006). Salvage of failed dynamic hip screw fixation of intertrochanteric fractures. Injury.

[13] Subash Y (2020). Valgus Osteotomy with DHS Fixation in the Management of Malunited Intertrochanteric Fractures in a Rural Population. Malays Orthop J.

[14] Saha P, Ayan S, Bandyopadhyay U, Mukhopadhyay AS, Bhattyacharyya G, Mukhopadhyay KK (2015). Anatomical reconstruction of unstable trochanteric fractures through posterior approach. J Orthop Allied Sci.

[15] Chen H, Tang P. A medial buttress construct and a fracture fixation device for femoral neck. U.S. patent 11,000,320 B2. 2021 May 11. https://worldwide.espacenet.com/searchResults?ST=singleline&locale=en_EP&submitted=true&DB=&query=US11000320&Submit=Search.

[16] Hoppenfeld S, deBoer P, Buckley R (2021). Surgical exposures in orthopaedics : the anatomic approach [M].

[17] Rose MS, Koshman ML, Spreng S, Sheldon R (1999). Statistical issues encountered in the comparison of health-related quality of life in diseased patients to published general population norms: problems and solutions. J Clin Epidemiol.

[18] Nherera L, Trueman P, Horner A, Watson T, Johnstone AJ (2018). Comparison of a twin interlocking derotation and compression screw cephalomedullary nail (InterTAN) with a single screw derotation cephalomedullary nail (proximal femoral nail antirotation): a systematic review and meta-analysis for intertrochanteric fractures. J Orthop Surg Res.

[19] Lambers A, Rieger B, Kop A, D'Alessandro P, Yates P (2019). Implant Fracture Analysis of the TFNA Proximal Femoral Nail. J Bone Joint Surg Am.

[20] Lotzien S, Rausch V, Schildhauer TA, Gessmann J (2018). Revision of subtrochanteric femoral nonunions after intramedullary nailing with dynamic condylar screw. BMC Musculoskelet Disord.

[21] Nie SB, Zhao YP, Li JT, et al. Medial support nail and proximal femoral nail antirotation in the treatment of reverse obliquity inter-trochanteric fractures (Arbeitsgemeinschaft fur Osteosynthesfrogen/Orthopedic Trauma Association 31-A3.1): a finite-element analysis. Chin Med J (Engl). 2020;133(22):2682–2687. doi:10.1097/CM9.0000000000001031 10.1097/CM9.0000000000001031 PMC7647506 32889910

[22] Nie S, Li M, Ji H, Li Z, Li W, Zhang H (2020). Biomechanical comparison of medial sustainable nail and proximal femoral nail antirotation in the treatment of an unstable intertrochanteric fracture. Bone Joint Res.

[23] Zhang W, Antony Xavier RP, Decruz J, Chen YD, Park DH (2021). Risk factors for mechanical failure of intertrochanteric fractures after fixation with proximal femoral nail antirotation (PFNA II): a study in a Southeast Asian population. Arch Orthop Trauma Surg.

[24] Qi L, Zhang W, Chen H. Treatment of ipsilateral femoral neck and shaft fracture by augmented fixation via modified anterior approach: A case report. Trauma Case Rep. 2022;39:100650. Published 2022 Apr 27. doi:10.1016/j.tcr.2022.100650 10.1016/j.tcr.2022.100650 PMC9092491 35571578

[25] Chen H, Li J, Chang Z, Liang X, Tang P. Treatment of femoral neck nonunion with a new fixation construct through the Watson-Jones approach. J Orthop Translat. 2019;19:126–132. Published 2019 Apr 28. doi:10.1016/j.jot.2019.04.004 10.1016/j.jot.2019.04.004 PMC6896481 31844620

[26] Li J, Yin P, Zhang L, Chen H, Tang P (2019). Medial anatomical buttress plate in treating displaced femoral neck fracture a finite element analysis. Injury.

[27] Buckley ER, Moran GC, Apivatthakakul T (2018). AO Principles of Fracture Management.

